# Generation of skin tone and pigmented region‐modified images using a pigment discrimination model trained with an optical approach

**DOI:** 10.1111/srt.13486

**Published:** 2023-09-28

**Authors:** Geunho Jung, Semin Kim, Jongha Lee, Sangwook Yoo

**Affiliations:** ^1^ AI R∖&D Center lululab Inc. Seoul Republic of Korea

**Keywords:** deep learning, melanin index, pigmented region, skin tone

## Abstract

**Background:**

Skin tone and pigmented regions, associated with melanin and hemoglobin, are critical indicators of skin condition. While most prior research focuses on pigment analysis, the capability to simulate diverse pigmentation conditions could greatly broaden the range of applications. However, current methodologies have limitations in terms of numerical control and versatility.

**Methods:**

We introduce a hybrid technique that integrates optical methods with deep learning to produce skin tone and pigmented region‐modified images with numerical control. The pigment discrimination model produces melanin, hemoglobin, and shading maps from skin images. The outputs are reconstructed into skin images using a forward problem‐solving approach, with model training aimed at minimizing the discrepancy between the reconstructed and input images. By adjusting the melanin and hemoglobin maps, we create pigment‐modified images, allowing precise control over changes in melanin and hemoglobin levels. Changes in pigmentation are quantified using the individual typology angle (ITA) for skin tone and melanin and erythema indices for pigmented regions, validating the intended modifications.

**Results:**

The pigment discrimination model achieved correlation coefficients with clinical equipment of 0.915 for melanin and 0.931 for hemoglobin. The alterations in the melanin and hemoglobin maps exhibit a proportional correlation with the ITA and pigment indices in both quantitative and qualitative assessments. Additionally, regions overlaying melanin and hemoglobin are demonstrated to verify independent adjustments.

**Conclusion:**

The proposed method offers an approach to generate modified images of skin tone and pigmented regions. Potential applications include visualizing alterations for clinical assessments, simulating the effects of skincare products, and generating datasets for deep learning.

## 1 INTRODUCTION

Skin tone and pigmented regions related to the concentration and distribution of skin pigments. They serve as important indicators of an individual's skin condition^1^, ^2^, ^3^ and play important roles in skincare products and diagnostics.^4^, ^5^ However, detecting subtle variations in these attributes can be challenging for the naked eye, potentially leading to subjective interpretations. Therefore, clinical devices like Mexameter for point measurement^6^ and imaging devices such as VISIA and CSKIN systems for analyzing spatial distribution information are often employed.^7^ Several methods have been proposed for skin pigment analysis, including deriving the melanin and erythema indices through a formula composed of RGB channel intensities,^8^ estimating skin chromophore concentrations through spectral information measured in diffuse reflectance spectroscopy^9^ and hyperspectral imaging,^10^ or acquiring different modulated light images in spatial frequency domain imaging.^11^

Beyond analysis, simulating various pigment conditions can greatly broaden potential applications. These applications encompass visualizing changes for clinical purposes, simulating skincare product effects, and generating deep learning datasets for early disease detection. However, most prior research has primarily focused on pigment analysis for diagnostic purposes, with only a few studies generating pigment‐modified images as application examples.^12^, ^13^ A common approach is to use independent component analysis (ICA) to transform the RGB channels of skin images into melanin, hemoglobin, and shading axes, and then modify the pigment information to change the skin tone.^12^ Existing techniques for pigment modification can numerically control pigmentation levels for image generation, but they have several limitations: they involve repeating mathematical operations such as least‐square fitting on all pixels,^14^ which is computationally intensive and memory‐demanding. Furthermore, these techniques require a wide range of pigment concentration variation within an image for applying principal component analysis (PCA) or ICA, which may necessitate additional work to find suitable areas by repeatedly sliding a window across the face.^12^

Recently, deep learning‐based methods, notably generative adversarial networks (GAN), have been employed to generate diverse skin images in the computer graphics field.^15^, ^16^, ^17^ Most of this research focuses on style transfer that combines the features of two face images, while only a few studies transform the skin tone by roughly adjusting the darkness of the masked area.^18^ However, these approaches require a large number of face images for training, and it is challenging to numerically control the degree of skin tone transformation with distinguishing melanin and hemoglobin contents. Moreover, the generative models can unintentionally modify or generate features, such as creating pigmented regions that were not present in the original image. Also, the resolution of images generated by these models is often limited by available computing resources, making them unsuitable for skin analysis.

In this study, we propose a new method that combines the optical approach with deep learning techniques to create numerically controlled skin tone and pigmented region‐modified images. The pigment discrimination model takes skin images as inputs, generating melanin, hemoglobin, and shading maps as outputs. By solving the forward problem, we reconstruct the outputs into skin images, and the model is trained by minimizing the discrepancy between the reconstructed and input images. We then modify the melanin and hemoglobin maps and reconstruct them into pigment‐modified images. This process enables us to numerically control the degree of change in skin tone or pigmented regions for melanin and hemoglobin, respectively. We quantify the changes in skin tone using the individual typology angle (ITA) method^19^, ^20^, ^21^ and levels of pigmented regions using the melanin and erythema indices^8^ to ensure intended pigmentation alterations.

## 2 METHODS

**FIGURE 1:**
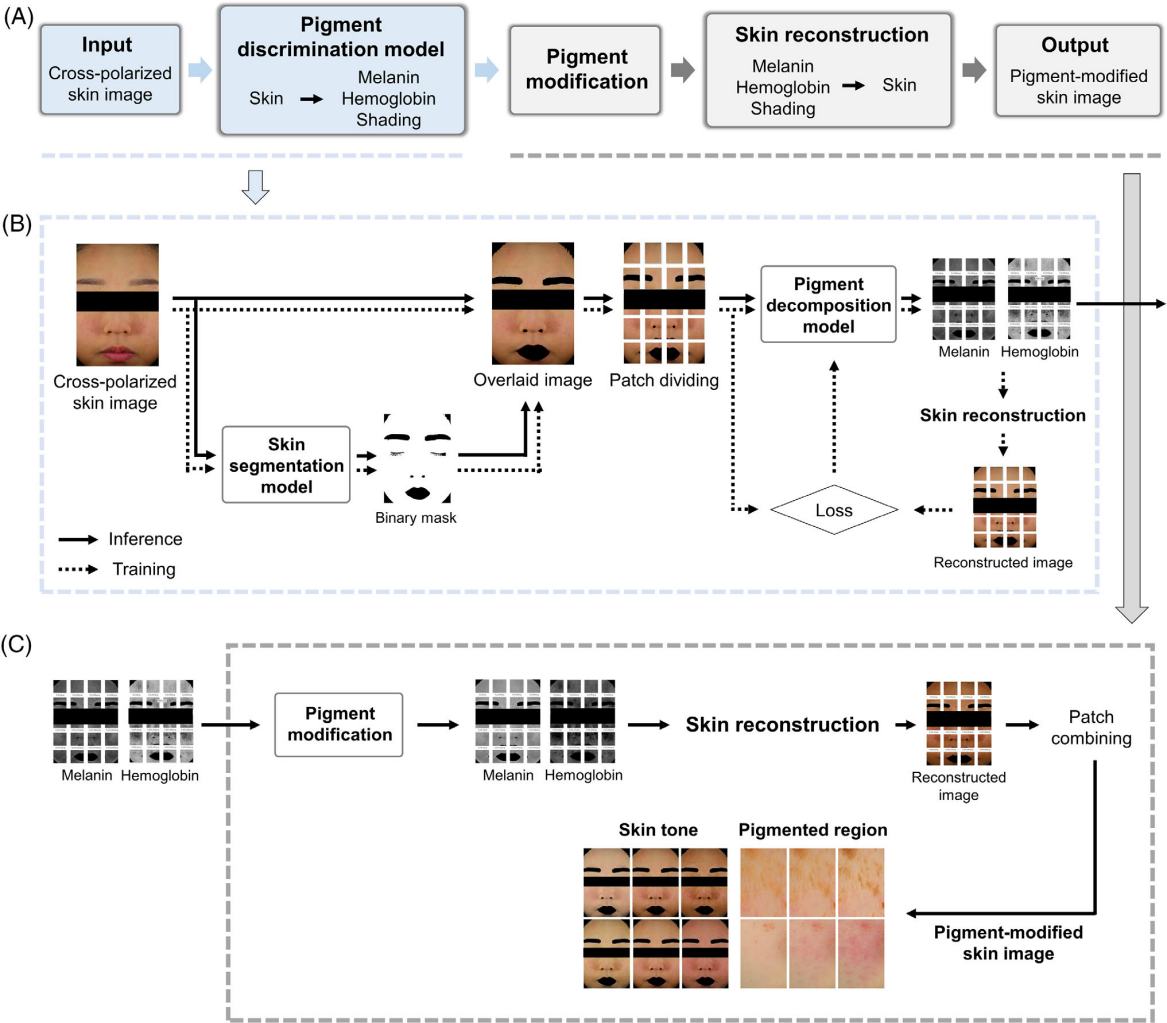
Schematic diagram illustrating the entire process of training the pigment discrimination model and generating pigment‐modified images. (A) Cross‐polarized skin images are decomposed into melanin, hemoglobin, and shading maps by the pigment discrimination model. The melanin and hemoglobin maps are altered to reconstruct pigment‐modified images. (B) The training and inference process of the pigment discrimination model. In the pre‐processing step, a binary mask is created using a pre‐trained skin segmentation model to separate the skin region and is overlaid on the input image. The overlaid image is divided into multiple patches and fed into the pigment discrimination model. The melanin, hemoglobin, and shading maps are generated as outputs and are reconstructed into skin images by solving the forward problem. The training process involves a loss function designed to minimize the discrepancy between the reconstructed image and the input image. For inference, the output maps are forwarded to the next step. (C) The process of generating pigment‐modified images. Depending on the intended variation of skin tone or pigmented regions, the melanin and hemoglobin maps are altered, and their reconstructed images are combined to form a full‐sized pigment‐modified image.

The overall process for generating a pigment‐modified image is depicted in Figure 1. The method involves extracting pigment maps from the input images, modifying the melanin and hemoglobin maps based on the intended variations of skin tone or pigmented regions, and subsequently reconstructing pigment‐modified images by solving the forward problem. We quantify the degree of changes in the modified image using the ITA for skin tone, as well as the melanin and erythema indices for pigmented regions. These estimated values are then compared with the intended degree of change to evaluate the performance of the proposed approach.

### 2.1 Pigment discrimination model

In our previous research,^22^ we employed the skin decomposition principle from the field of computer graphics^23^ to train a skin analysis model for dermatological research. From general skin images, this model produces four outputs: melanin, hemoglobin, shading, and specular maps, and yields results comparable to those of clinical diagnostic equipment. To further enhance the model, we introduced several modifications. Similar to most skin pigment analysis tools, we limited the input to cross‐polarized images, simplifying the model structure by eliminating the specular component from the outputs. We transitioned model architecture from the basic UNet to the more advanced ResUNet++,^24^ leading to improved performance and shortened training time. In this study, we utilized the revised training framework and the ResUNet++ model structure. Additionally, in our previous research,^25^ we confirmed that applying ground truth for the pigments could further improve performance. However, given the considerable usability benefits of using the optical method in place of the ground truth, we chose not to use ground truth for the pigments in this study.

#### 2.1.1 Training and inference framework

The training and inference framework for the pigment discrimination model is depicted in Figure 1B. During pre‐processing, to facilitate model training based on skin optical properties, a binary mask is created to distinguish skin from non‐skin areas and overlaid on the image. This task is accomplished using a skin segmentation model constructed with the ResUNet++ architecture. This model, trained on 2100 skin images with manually prepared ground truths and accepting an input size of 640 × 480 pixels, achieved a complete intersection over union (CIoU)^26^ score of 0.961 on a test set of 232 images.

Subsequently, the input image is divided into multiple patches, each integrating a 15 pixel overlap at its boundaries. The outputs of these patches are combined with applying an inverse gradient to the overlapping regions. This strategy serves to mitigate computational and memory constraints related with the model's input dimensions and ensures minimized potentially visible boundary lines in the combined image. For this study, the training images were divided into 310 × 310 pixel patches and then randomly cropped to 256 × 256 pixels for augmentation purposes. In contrast, the test set was directly divided into 256 × 256 pixel patches.

Lastly, to restore the linear attributes of color, an inverse gamma correction is applied, converting the color space from standard RGB (sRGB) to linear RGB.

The pigment discrimination model produces melanin, hemoglobin, and shading maps, each sized 256 × 256 pixels. These outputs are then reconstructed into a skin image. The model aims to produce outputs that approximate the actual skin pigment conditions of the input image, resulting in a reconstructed image that closely resembles the input image. To minimize the discrepancy between the input and the reconstructed image, the mean squared error (MSE) and peak signal‐to‐noise ratio (PSNR) are employed as loss functions during training and evaluation. Moreover, a modified version of the ResUNet++ structure^24^ is utilized, where the number of channels is halved to alleviate memory demands.

#### 2.1.2 Skin reconstruction

**FIGURE 2:**
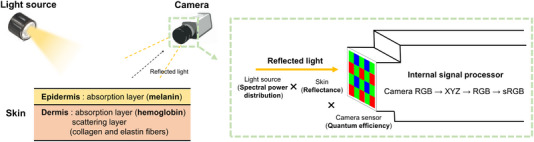
Skin image formation process consists of the following steps: illuminated light is reflected from the skin, detected by the camera, and processed to form a skin image. The skin layers are optically modeled as absorption layers (due to melanin and hemoglobin) and a scattering layer (due to collagen and elastin fibers), contributing to the skin's reflectance. The skin image is formed in the camera‐specific RGB color space by multiplying the spectral power distribution of the light source, the skin reflectance, and the camera sensor's quantum efficiency. Finally, the camera's internal signal processor converts the color space of the image to standard RGB.

The general skin image formation process is depicted in Figure 2. Illuminated light is partially specularly reflected from the skin surface following Fresnel's law,^27^ while the remaining light is absorbed and scattered within the skin layers before being diffusely reflected outwards. The overall reflectance of the skin is determined by absorption due to the main chromophores, melanin and hemoglobin, and by scattering, which includes both Rayleigh and Mie scattering.^28^ Both specularly and diffusely reflected light are detected by the camera sensor to form a skin image. In this study, we utilize cross‐polarized images to analyze the pigment information within the diffusely reflected light. Through these processes, an image is created in the camera‐specific RGB color space. It is then sequentially converted to the CIE 1931 color space (XYZ), linear RGB, and finally to the standard RGB (sRGB) color space by the camera's internal signal processor.

The equations defining the absorption and reduced scattering coefficients are given below^29^:

(1) $$\mu_{a}\;\left(\lambda \right)=\Sigma_{i}\;V_{i}\in_{i}\left(\lambda \right)C_{i}$$

(2) $$\mu_{s}^{\prime }\;\left(\lambda \right)=\;a\left(f_{\mathrm{Ray}}{\left(\frac{\lambda }{\lambda_{0}}\right)}^{-4}+\left(1-f_{\mathrm{Ray}}\right){\left(\frac{\lambda }{\lambda_{0}}\right)}^{-b_{\mathrm{Mie}}}\right)$$

The absorption coefficient, $\mu_{a}\left(\lambda \right)$, represents the light absorption by chromophores, *i* (melanin, hemoglobin). Each chromophore's absorption is calculated by multiplying its volume fraction $V_{i}$, extinction coefficient **∈** ***_i_*** , and concentration $C_{i}$. This study sets the wavelength λ to range from $\lambda_{min}=\;450$ nm to $\lambda_{max}=\;750$ nm. The reduced scattering coefficient, $\mu_{s}^{\prime }\left(\lambda \right)$, consists of the scattering amplitude *a*, scattering power *b*, and the fraction of the Rayleigh scattering events $f_{Ray}$, thus incorporating both Rayleigh and Mie scattering effects.

Assuming that the majority of the diffusely reflected light is reflected from the dermis layer, the skin reflectance can be expressed as follows, based on the Kubelka‐Munk theory^30^:

(3) $$R\;\left(\lambda \right)=T_{\mathrm{epidermis}}\;{\left(\lambda \right)}^{2}R_{\mathrm{dermis}}\left(\lambda \right)$$

The skin reflectance, R(λ), is characterized by the path of light from illumination to reflection from the dermis layer: the fraction of light transmitted through the epidermis layer $T_{epidrmis}\left(\lambda \right)$, the fraction of light reflected from the dermis layer $R_{dermis}\left(\lambda \right)$, followed by an additional multiplication by $T_{epidermis}\left(\lambda \right)$. Using this approach with the conditions of volume fractions for melanin and hemoglobin range from 1.3% to 43% and from 2% to 7%, respectively,^31^, ^32^ we have applied the reflectance values used in previous studies^23^, ^33^ to our research.

The equations for skin image reconstruction are as follows:

(4) $$\;I_{m}=\int_{\lambda_{\min}}^{\lambda_{\max}}L\left(\lambda \right)\cdot R\left(\lambda \right)\cdot M_{\mathrm{shading}}\cdot C_{m}d\lambda \;$$

(5) $$\;I_{\mathrm{RGB}}=T_{\mathrm{XYZ}\;\mathrm{to}\;\mathrm{RGB}}\;\cdot T_{\mathrm{cam}\_\mathrm{RGB}\;\mathrm{to}\;\mathrm{XYZ}}\cdot \left(M_{\mathrm{wb}}\cdot I_{\mathrm{cam}\_\mathrm{RGB}}\right)$$

The image $I_{cam}=\left[I_{r},I_{g},I_{b}\right]\;$ (each channel *m* in *r*, *g*, and *b*) is formed in a device‐dependent color space by integrating the product of the spectral power distribution (SPD) of the light source L(λ), the skin reflectance R(λ), the shading map $M_{shading}$, and the quantum efficiency of the camera sensor $C_{m}$ across in defined wavelength range. The RGB values of this image are then normalized by the those of directly detected from the light source to the camera, $M_{wb}$, thereby achieving white balance. The image is then sequentially converted to common color spaces, XYZ and RGB, by multiplying by the corresponding matrix *M*. During the inference process, gamma correction is additionally applied to the image $I_{RGB}$ to convert it into the standard RGB (sRGB) color space.

### 2.2 Individual typology angle

**FIGURE 3:**
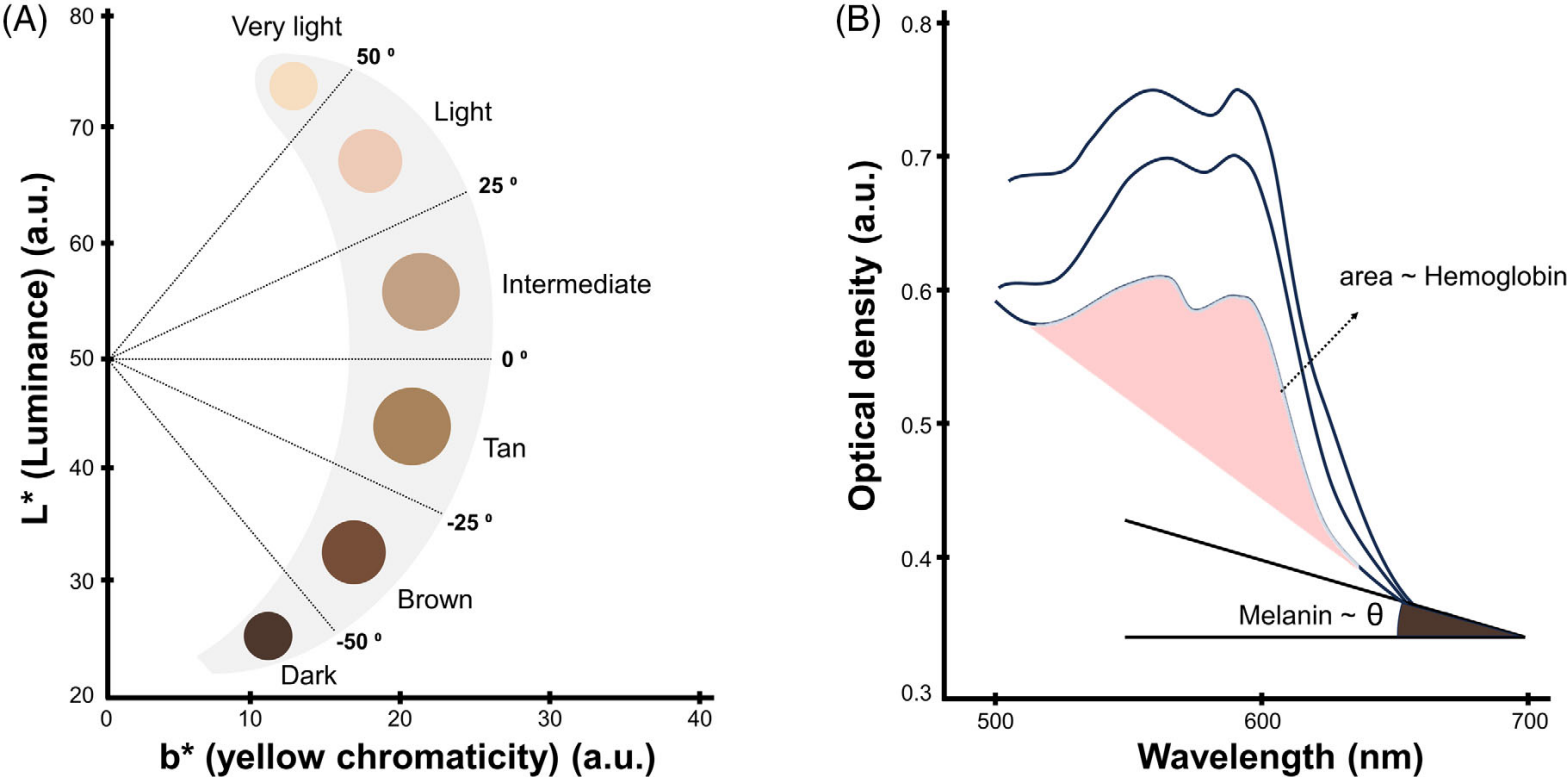
Illustration of the principles for quantifying skin tone and pigmentation levels. (A) The ITA is a method used to analyze skin tone. It involves converting to the Lab* space, followed by calculating the angle formed by the L* coordinate (lightness) and b* coordinate (blue to yellow). (B) The slope of optical density in the spectral range of 620 to 720 nm is directly proportional to melanin content, while changes in hemoglobin content alter absorption in the 560 to 650 nm spectral range. These characteristics form the basis for defining melanin and erythema indices.^34^ ^.^

In this study, we quantified skin tone using the ITA method as illustrated in Figure 3A, following the steps outlined in.^19^ Initially, a binary mask created by the skin segmentation model is applied to determine the average RGB value of the skin area, which is then converted into the Lab* color space. Here, *L* denotes lightness, while *a* and *b* represent the color dimensions transitioning from green to red and blue to yellow, respectively. The angle formed by the *L* and *b* coordinates, using L=50 as the reference, is calculated as follows:

(6) $$\mathrm{ITA}\;=\arctan\left(\frac{L-50}{b}\right)\;\times \frac{180^{\circ }}{\pi }$$

In this equation, the outcome is converted from radian to degree by multiplying with $\frac{180^{\circ }}{\pi }$. The ITA values can further categorize skin tones from ‘dark’ to ‘very light’ (as shown in Figure 3A), though this categorization is not the main emphasis of our study. Moreover, even for the same subject, skin color may exhibit variations depending on factors like the light source and camera models employed. As such, the ITA analysis in our research was conducted using images captured with consistent equipment.

### 2.3 Melanin and erythema index

Figure 3B illustrates the principles behind the quantification of melanin and hemoglobin contents. Optical density (OD) is utilized as a representative measure of light absorption. It is derived from the logarithm of reflectance, which is the ratio between the reflected light intensity *I* and the illuminated light intensity *I*_0_:

(7) $$\mathrm{OD}\;=\;-\log\left(\frac{I}{I_{0}}\right)$$

Although melanin exhibits high absorption in the ultraviolet (UV) range, applying this property to common visible light sources and cameras is challenging. Previous research suggests that the slope of OD between 620 and 720 nm, which corresponds to the red channel of most camera sensors, is primarily influenced by melanin content and less by hemoglobin.^35^ Hence, the OD of the red channel is used as a simplified melanin index (M.I) for color images:

(8) $$M.I\;=\;OD_{R}$$

Variations in hemoglobin content predominantly influence the OD within the 510–610 nm wavelength range. Utilizing this characteristic, the erythema index is determined from spectra acquired with a spectrometer to quantify hemoglobin presence.^34^ For color images, the erythema index, $E.I_{1}$, is calculated using the OD difference between the green channel (560 nm) and the red channel (650 nm). These channels correspond to relatively higher and lower hemoglobin absorption, respectively. This approach is termed Diffey's method.^36^ To compensate for possible interference from melanin absorption on the erythema index, an alternative version, $E.I_{2}$, incorporates intensities from all RGB channels, following Jacovels' method^37^:

(9) $$E.\;I_{1}=\;OD_{G}-OD_{R}$$

(10) $$E.\;I_{2}=\frac{\sqrt{I\left(B\right)\cdot I\left(R\right)}}{I\left(G\right)}\;$$

In this study, we adopted these color image‐based methodologies to assess alterations in skin tone using the ITA approach. Additionally, we examined pigmentation levels by means of both the melanin and erythema indices (employing Diffey's and Jacovels' methods). The results are compared with variations altered on pigment maps, confirming the intended changes in pigmentation.

## 3 EXPERIMENT

We obtained cross‐polarized images, along with corresponding melanin and hemoglobin distribution maps, for 104 subjects from the global medical research center. These images were captured using the VISIA‐CR acquisition system (Canfield Scientific Inc.) and subsequently analyzed with the VISIA VAESTRO image analysis system's RBX‐Brown and RBX‐Red Processing 1.0 modules.^38^ Given that this system has been widely recognized for research and diagnostic purposes,^39^, ^40^ we selected its results as our reference to validate our model's performance. Data acquisition for all subjects complied with national regulations and institutional policies on research ethics. Prior informed consent was obtained from all participants, and this study received approval from the authors' institutional review board.

To train the pigment discrimination model, we divided the subjects into a training set (74 subjects) and a test set (30 subjects). A detailed discussion about the dataset sizes is provided in the discussion (Section 4). Images in the training set were resized to 1240 × 1550 pixels, divided into 310 × 310 pixel patches, and randomly cropped to 256 × 256 pixels for augmentation. Conversely, the test set images were resized to 979 × 1461 pixels and divided into 256 × 256 pixel patches. The conditions for image acquisition were set to the spectral power distribution of the ‘D65’ standard illuminant and the quantum efficiency of the ‘Canon 5D Mark II’ camera model.^41^ Computations were performed using an RTX 3090 GPU on the PyTorch platform. Image augmentation utilized the albumentations library,^42^ and training lasted around 16 h, reaching 800 epochs. Training was guided by the MSE loss, while the PSNR served as the evaluation metric. We validated the pigment discrimination capability of the trained model by comparing its correlation coefficients^43^ to those derived from the melanin and hemoglobin maps provided by the VISIA system.

Using the same test set of 30 subjects, we produced skin tone‐modified images. The modifications were achieved by uniformly adjusting all pixel values in the melanin and hemoglobin maps, ranging from −1 to +1 in arbitrary units, incremented by 0.2. We then computed the average ITA value for the skin‐segmented regions across all subjects and compared it to the added values.

Regarding pigmented regions, given our dataset primarily comprised of non‐patient individuals, finding out regions of hyperpigmentation indicative of a disease proved challenging. We addressed this by selecting areas with relatively more pigmentation than their adjacent normal skin as our test set. To avoid potential data redundancy, especially when multiple similar regions from a single subject were considered, we ensured that only one region per subject was included. This resulted in ten datasets each for melanin and hemoglobin. Pigmented region‐modified images were generated by scaling the pixel values of melanin and hemoglobin maps uniformly. Utilizing custom‐designed binary masks, we evaluated the difference in pigment indices between the pigmented region and the surrounding normal skin, contrasting this with the scaling factor. The scaling values for melanin spanned from 0.8 to 1.4 (incremented by 0.1), while for hemoglobin they ranged between 0.6 and 1.4, both in arbitrary units. These ranges were chosen to ensure the generated images appeared natural, avoiding any excessive changes.

Our method's efficacy was validated through quantitative comparisons concerning skin tone and pigmented regions. Additionally, demonstrating the overlaid regions of melanin and hemoglobin provided clear evidence of independent modification.

## 4 RESULTS

**FIGURE 4:**
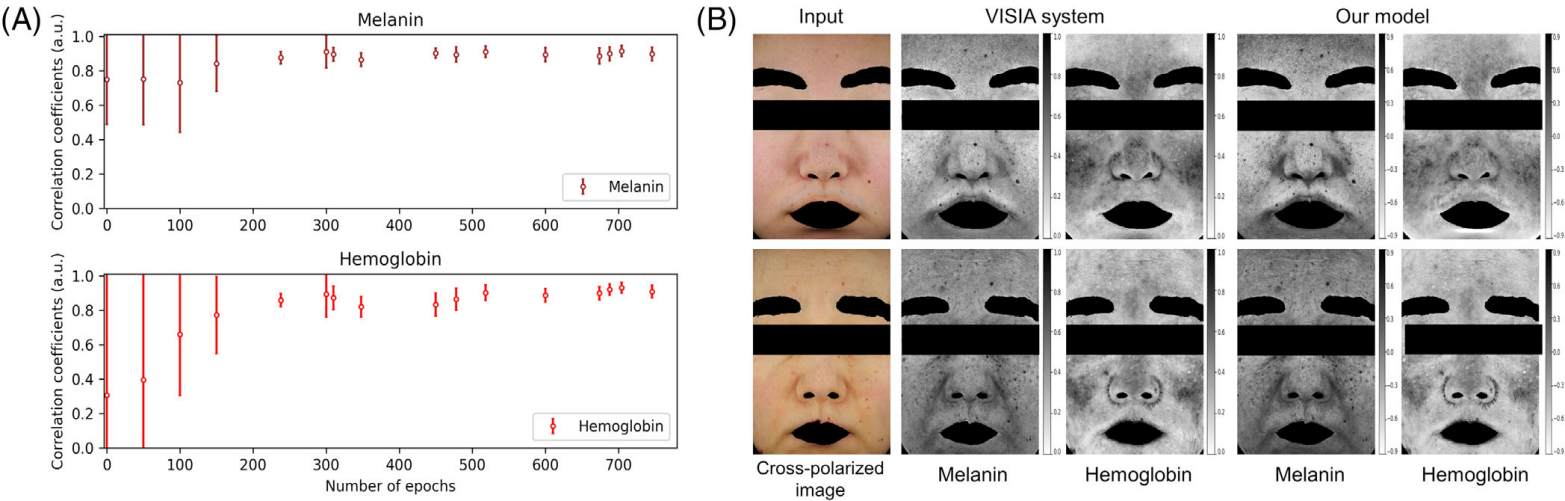
Assessing the performance of the pigment discrimination model compared to the VISIA system. (A) The correlation coefficients of the (upper) melanin and (lower) hemoglobin maps with the results from the VISIA system. The model was trained for 800 epochs, and the sampled models were applied to the test set (30 subjects) through post‐processing. (B) The melanin and hemoglobin maps from the (second and third columns) VISIA system and (fourth and fifth columns) our model are presented for comparison.

The performance of the pigment discrimination model was evaluated by comparing it with the VISIA system, as illustrated in Figure 4. Throughout the training, models that achieved a PSNR greater than 35 dB between the input and reconstructed images were saved. A subset of these saved models was sampled and applied to the test set, which comprised 30 subjects. The model trained to 704 epochs achieved a PSNR of 43.25 dB. This model showed the highest correlation coefficients for melanin and hemoglobin, values of 0.915 (std 0.033) and 0.931 (std 0.032), respectively. The output maps of this model and the VISIA system are presented in Figure 4B. Since the VISIA system's results were color images (brown for melanin and red for hemoglobin maps) that represented different concentrations, we converted these results to grayscale for a consistent comparison.

**FIGURE 5:**
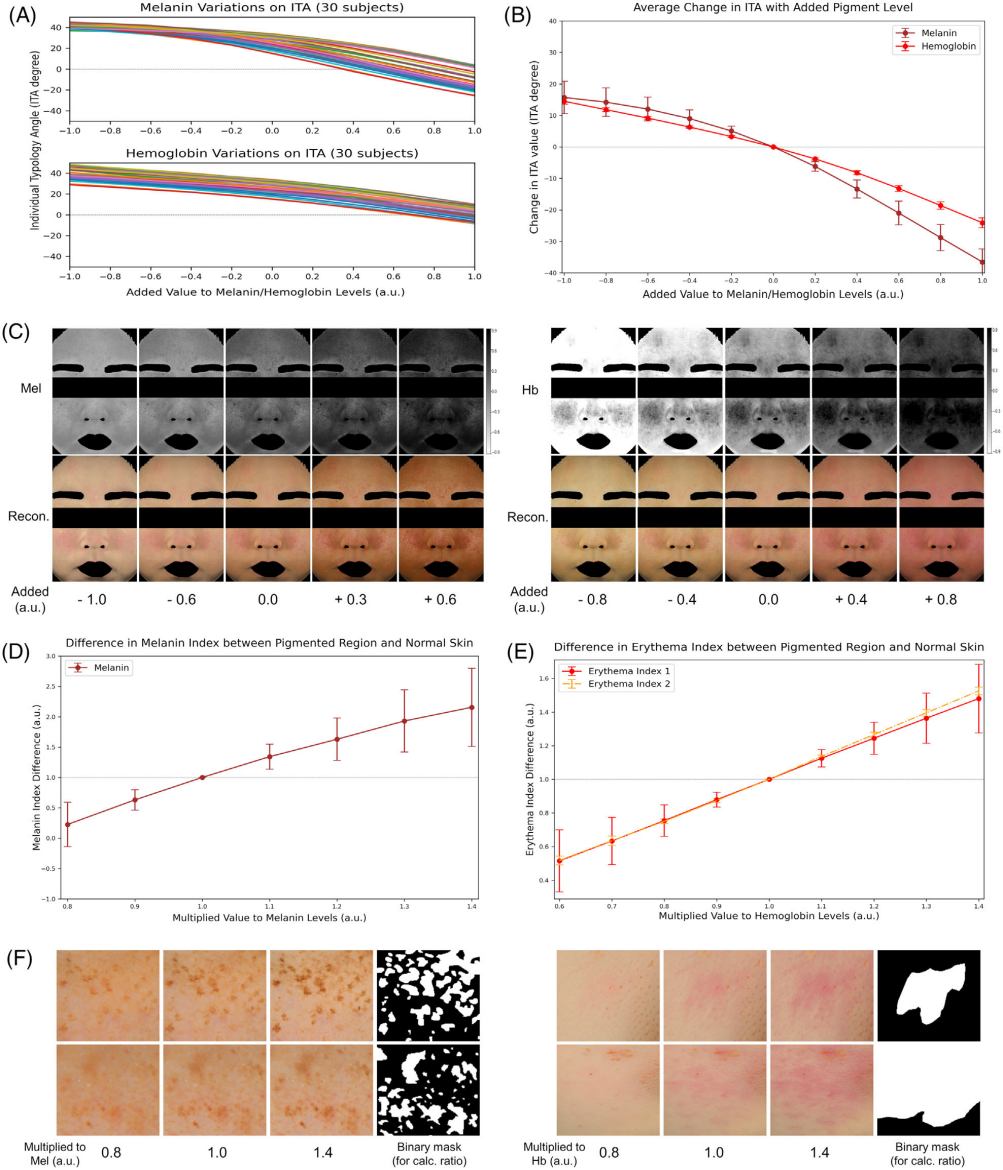
Quantitative comparisons were made for skin tone (ITA) and pigmented regions (melanin and erythema indices) with altered melanin and hemoglobin levels, and the corresponding modified images are presented. (A) The ITA values for 30 subjects, and (B) the mean change in ITA values are depicted with added values to melanin and hemoglobin maps. (C) The upper row shows individually modified melanin (left) and hemoglobin (right) maps, with their corresponding skin tone‐modified images displayed in the lower row. For the pigmented regions modification, the (D) melanin index and (E) erythema index differences are calculated between the pigmented region and normal skin, and compared with the multiplied values to melanin and hemoglobin maps. The erythema index is used as a measure to quantify hemoglobin content, and two different methods are applied in this study.^36^, ^37^ (F) The pigmented region‐modified images were reconstructed by multiplying factors of 0.8 and 1.4 to the melanin (left) and hemoglobin (right) maps, respectively. The fourth column in each set displays a manually designed binary mask employed for the computation of the pigment index ratio.

The quantified changes in skin tone (ITA) and pigmented regions (melanin and erythema indices) were compared with the altered values of melanin and hemoglobin levels in Figure 5. For skin tone, the ITA values for 30 subjects and the mean change in ITA based on the added value to melanin and hemoglobin maps were presented. For the pigmented regions, we analyzed the differences in the melanin and erythema indices between the pigmented regions and normal skin against the multiplied values in the melanin and hemoglobin maps. The differences in melanin and erythema indices, representing the relative contrast between pigmented and normal skin areas, were computed using manually designed binary masks. Additionally, the modified images for both skin tone and pigmented regions were displayed.

## 5 DISCUSSION

The performance of the pigment discrimination model is both quantitatively and qualitatively evaluated, as depicted in Figure 4. Since the training framework does not use ground truth for pigment maps, the model's ability to distinguish pigment maps is assessed through post‐processing. This is done by comparing correlation coefficients with those from the VISIA system. The graph of correlation coefficients suggests a stabilization of training after roughly 250 epochs, with minimal changes in the standard deviation after 500 epochs (Figure 4A). At 704 epochs, the model exhibits the highest correlation coefficients for melanin and hemoglobin: 0.915 (std 0.033) and 0.931 (std 0.032), respectively. The PSNR value of this model (43.25 dB) indicates its capability to closely resemble input images, as a PSNR value above 30 dB typically exceeds human discernment.^44^, ^45^ The melanin and hemoglobin maps generated by our model are compared with those from the VISIA system in Figure 4B, showing similar trends. Additionally, we compared subjects of different darkness degrees and found that the melanin map values in the lower row are generally higher than those in the upper row.

Figure 5 presents the skin tone and pigmented region‐modified images, derived from the altered outputs of the pigment discrimination model. We employed a direct approach, adding identical values to the melanin and hemoglobin maps to shift the overall skin tone, whereas multiplying by identical values enhances the contrast between the pigmented regions and normal skin. Conventional color image‐based methodologies were then utilized to quantify skin tone (ITA) and pigmentation levels (melanin and erythema indices), to examine their relationship with the modified values. The ITA graph in Figure 5A should be interpreted alongside the ITA‐based skin color (Figure 3) and the fabricated skin tone‐modified images (Figure 5C). While this study uses both melanin and hemoglobin to adjust skin tone, the ITA method deploys a singular variable (angle) to represent the overall skin tone. Looking at the color perspective (Figure 3), significant changes in skin darkness are observed across most of the ITA range. Especially in the ‘light’ and ‘very light’ categories with the elevated ITA values, redness shifts are also discernible. Melanin, known for its extensive light absorption across wavelengths, heavily affects skin darkness.^46^ Conversely, hemoglobin, which has high absorption in the green wavelength, accentuates the complementary red color.^47^ Additionally, a modified method that incorporates the a* value into the ITA^48^ might be anticipated to be more sensitive to hemoglobin. Nonetheless, in this study, we chose the widely accepted and more popular approach, ITA.

In Figure 5A, it is evident that the addition of negative values up to −1.0 results in the ITA value rising to roughly 40 to 50. This tendency suggests that excessive reductions in either melanin or hemoglobin values make the skin tone towards a much lighter color, as depicted in Figure 5C. Moreover, the variation at this value (−1.0) is wider than that of melanin. This denotes that as hemoglobin quantities diminish, melanin's influence on the ITA might grow proportionally stronger, and vice versa. This is because the ITA is more sensitive to variations in skin darkness, which lead to a broader distribution when hemoglobin levels decrease. Conversely, with the value's increment to +1.0, the ITA decrease is more profound for melanin (around −30) than for hemoglobin (around −10). This trend also shows that the change in ITA for melanin exceeds that for hemoglobin at this value (+1.0). This behavior can be attributed to the ITA's sensitivity to skin darkness, which is closely correlated with melanin content. Also, the ITA value change ascends to approximately 15 for both melanin and hemoglobin cases when the added value is −1.0 (Figure 5B). As the ITA value saturates around 45–50 when one pigment is markedly diminished and the majority of the test set is concentrated around the value of 30, this difference of roughly 15 is anticipated. Figure 5C presents the skin tone‐modified images, which are displayed within a range defined to prevent excessive saturation in pigment maps. Considering that the absolute value of the hemoglobin map is relatively lower than that of melanin in the original image, many areas approach saturation (close to 0) when the added value is −0.8.

The enhancement of pigmented regions is more noticeable when the pigmentation level significantly differs from the surrounding normal skin, rather than measuring the level of the region itself. We therefore investigated the pigment index difference between the pigmented and normal skin, resulting in a proportional relation to the multiplied values for both melanin and erythema indices (Figure 5D, E). While the erythema index changes with the multiplied value in accordance with hemoglobin levels, the melanin index exhibits a larger change compared to the multiplied value, and an increased standard deviation as the multiplied value deviates from 1.0. Several reasons could account for these observations. First, the melanin map's discrimination may not have been sufficient. The outputs of the pigment discrimination model, specifically the shading components, indicate darker regions, such as shadows caused by lighting. These darker regions have characteristics similar to melanin, making them challenging for the model to distinguish. Furthermore, the melanin index formula solely uses red channel information. Although hemoglobin's absorption is not as prominent as melanin's, it also influences the red color wavelength. Thus, relying solely on color images may present limitations. One potential solution is altering the melanin index formula. It could include information from the UV wavelength range or the front part of the visible light spectrum, captured using a multi‐spectral imaging system. Next, the erythema index is influenced by the multiplied value in both methods. However, the range of the standard deviation differs (Figure 5E). This variation occurs because erythema index 1, a simpler method, incorporates the effect of melanin content, while erythema index 2 is an improved approach that minimizes melanin's influence. Figure 5F shows images modified for melanin and hemoglobin contents, separately. For reference, we also included a manually designed binary image used to calculate the melanin index difference.

To train the pigment discrimination model, we utilized a dataset of 74 subjects, which was sufficient for several reasons. Models typically designed to segment specific regions, such as hyperpigmented areas across the entire face, are trained to locate the interest regions at the image level and thus demand an extensive dataset, whereas our model iteratively predicts pigment levels from each pixel in facial regions of the input image. In other words, our model is trained to discriminate pigment information at the pixel level, allowing for efficient learning from a limited dataset. Given the practical challenges in data acquisition, we divided each image into 20 patches, expanding our training dataset to 1480 patch images. The model's optical training methodology, assuming consistent light source and camera conditions for every pixel, enables the patch‐based strategy. Consequently, the dataset of 74 subjects was sufficient to train the model, yielding pigment maps comparable to those of the VISIA system, as demonstrated in our results.

**FIGURE 6:**
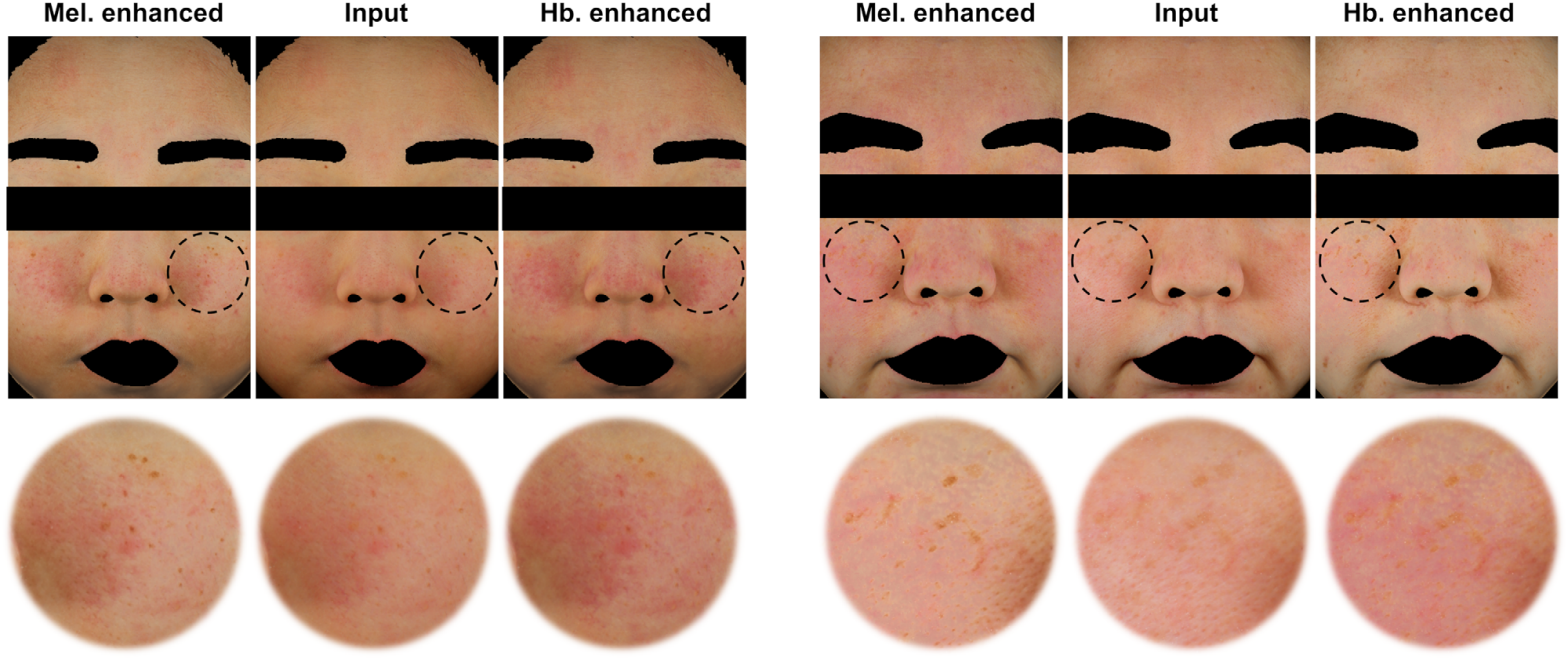
Images demonstrating the enhancement of individual pigments (hemoglobin and melanin) specifically within the overlaid pigmented regions, allowing for an analysis of their independent separation.

One notable advantage of our pigment discrimination approach over conventional GAN models is its ability to independently modify hemoglobin and melanin contents. Moreover, this modification is numerically controlled. This distinct advantage is exemplified in Figure 6, where regions overlaid with both pigments were identified and each pigment was individually enhanced. Furthermore, since our model is trained using an optical approach, it does not require ground truth for pigment maps. This versatility means our model can be applied across various image acquisition systems.

Despite the evident strengths of our model, its primary limitation lies in distinguishing between pigment maps, particularly for melanin. This limitation could be alleviated by incorporating the ground truth for shading maps and modifying the training framework while maintaining the process of skin reconstruction. If these improvements are made, our approach may produce more natural‐looking modified images even with substantial alterations in pigments. Additionally, there is potential for information saturation beyond the plotting range during model visualization. For instance, in Figure 5C, when the added value for hemoglobin is −0.8, the actual hemoglobin value could be zero, but it might also have been saturated due to the generally lower range of value distribution. Greater precision in controlling the plotting range of the pigment maps, potentially with a clinician's contact device, could minimize the amount of saturated information, thereby potentially enhancing performance. If these limitations are effectively addressed, the proposed approach holds promise for widespread application in skincare and diagnostics. For example, it could be utilized to generate training datasets or to create simulated images of pigmentation disorders for diagnostic purposes.

## 6 CONCLUSION

In this study, we introduced a method to generate skin tone and pigmented region‐modified images using a pigment discrimination model trained with an optical approach. This model, which takes a cross‐polarized image as input and outputs melanin, hemoglobin, and shading maps, represents a significant advancement in our ability to differentiate and modify skin pigmentation. A distinctive feature of our approach is its ability to numerically adjust these pigment maps, allowing for precise modifications of melanin and hemoglobin contents. We validated our approach by quantifying the skin tone of the generated images using the ITA and assessing pigmentation alterations using the melanin and erythema indices. The capacity to independently discern and manipulate melanin and hemoglobin content levels holds broaden potential applications. This includes not only skincare products but also diagnostic applications, significantly enhancing our understanding of skin conditions and our ability to use image‐based techniques for diagnosis and treatment.

## CONFLICT OF INTEREST STATEMENT

The authors have no relevant financial interests in this article and no potential conflicts of interest to disclose.

## Data Availability

The data associated with this study are not publicly available due to privacy and confidentiality considerations. They are only accessible for research purposes upon request to the corresponding author, Geunho Jung. Obtaining these data requires prior consent from the involved subjects and necessary approvals from the appropriate institutional committees at the author's institution.
